# Homœopathy and Rational Medicine

**Published:** 1901-02-23

**Authors:** R. E. Dudgeon

**Affiliations:** 63 Upper Berkeley Street


### Editor's Letter-Box.

[Our Correspondents are reminded that prolixity is a great bar to publication, and that brevity of style and conciseness of statement greatly facilitate early insertion.]

### HOMŒOPATHY AND RATIONAL MEDICINE.

To the Editor of *The Hospital*.

Sir,—Forgive me for thinking, when you spoke of the "beneficial effect of a certain drug discovered by a homœopathist, an old woman, or a naked savage," that that was equivalent to "taking the word" of these heterogeneous practitioners for their discovery of said beneficial effect; I confess I am yet unable to comprehend how otherwise you could learn that they had made such a discovery. But this may be owing to my own intellectual deficiencies, for have we not been told over and over again, by the partisans of the "rational medicine" we have abandoned for homœopathy, that we are either knaves or fools, or a combination of both? So, to my own foolish mind, it still appears that your "rational medicine" is merely a euphuism for "pure empiricism."

For saying that your school's employment of many of the remedies our school—we don't object to the word—has introduced into medicine is "a sort of homœopathy," you "leave me to the tender mercies of my fellow-homœopathists." I believe the latter will be tenderly merciful to me, as they all think as I do on that matter. Hahnemann had no opportunity of expressing his opinion on the subject, as he died before the partisans of "rational medicine" had begun to employ the remedies of his materia medica; but in the four first editions of his *Organon* he gives a long catalogue of cases treated by old-school physicians by medicines which he shows, on the authority of other old-school doctors, have the power to cause morbid states similar to those they cured; and this he calls "accidental or unconscious homœopathy," which is something more precise than "a sort of homœopathy." I may add that the fashionable practices of serum-therapy and opotherapy are also "a sort of homœopathy"—a bad sort, to be sure, but illustrations of the cure of diseases referable to the homœopathic formula "*similia similibus*."

Yours faithfully,

63 Upper Berkeley Street, R. E. DUDGEON.

February 13, 1901.

[This is mere trifling. Of course Hahnemann gives a list of drugs the use of which illustrates the "accidental or unconscious homœopathy" which, from his point of view, was at the bottom of all successful treatment by whomsoever administered; but that is a very different thing from considering that our use of many of the remedies introduced to medicine by homœopathists indicates a "leaning to homœopathy." We are sorry that Dr. Dudgeon has referred to the "knaves or fools" style of argument, in which we have no wish to join, so that we think the matter had better now rest where it is.—ED. THE HOSPITAL.]

